# Association of *STAT4* Polymorphism with Severe Renal Insufficiency in Lupus Nephritis

**DOI:** 10.1371/journal.pone.0084450

**Published:** 2013-12-27

**Authors:** Karin Bolin, Johanna K. Sandling, Agneta Zickert, Andreas Jönsen, Christopher Sjöwall, Elisabet Svenungsson, Anders A. Bengtsson, Maija-Leena Eloranta, Lars Rönnblom, Ann-Christine Syvänen, Iva Gunnarsson, Gunnel Nordmark

**Affiliations:** 1 Section of Rheumatology, Department of Medical Sciences, Uppsala University, Uppsala, Sweden; 2 Molecular Medicine, Department of Medical Sciences and Science for Life Laboratory, Uppsala University, Uppsala, Sweden; 3 Rheumatology Unit, Department of Medicine, Karolinska University Hospital and Karolinska Institutet, Stockholm, Sweden; 4 Section of Rheumatology, Department Clinical Sciences, Lund University, Lund, Sweden; 5 Rheumatology/AIR, Department of Clinical and Experimental Medicine, Linköping University, Linköping, Sweden; 6 Science for Life Laboratory, Uppsala University, Uppsala, Sweden; Innsbruck Medical University, Austria

## Abstract

Lupus nephritis is a cause of significant morbidity in systemic lupus erythematosus (SLE) and its genetic background has not been completely clarified. The aim of this investigation was to analyze single nucleotide polymorphisms (SNPs) for association with lupus nephritis, its severe form proliferative nephritis and renal outcome, in two Swedish cohorts. Cohort I (n = 567 SLE cases, n =  512 controls) was previously genotyped for 5676 SNPs and cohort II (n = 145 SLE cases, n = 619 controls) was genotyped for SNPs in *STAT4, IRF5*, *TNIP1* and *BLK.*

Case-control and case-only association analyses for patients with lupus nephritis, proliferative nephritis and severe renal insufficiency were performed. In the case-control analysis of cohort I, four highly linked SNPs in *STAT4* were associated with lupus nephritis with genome wide significance with p = 3.7×10^−9^, OR 2.20 for the best SNP rs11889341. Strong signals of association between *IRF5* and an *HLA-DR3* SNP marker were also detected in the lupus nephritis case versus healthy control analysis (p <0.0001). An additional six genes showed an association with lupus nephritis with p <0.001 (*PMS2, TNIP1, CARD11, ITGAM, BLK* and *IRAK1*). In the case-only meta-analysis of the two cohorts, the *STAT4* SNP rs7582694 was associated with severe renal insufficiency with p  = 1.6×10^−3^ and OR 2.22. We conclude that genetic variations in *STAT4* predispose to lupus nephritis and a worse outcome with severe renal insufficiency.

## Introduction

Lupus nephritis (LN) constitutes one of the main clinical challenges in patients with systemic lupus erythematosus (SLE) and is a cause of significant morbidity and mortality. LN occurs in 15–55% of patients with SLE with the higher incidence in Asian and African populations [1]. Proliferative glomerulonephritis, classes III/IV, is considered to be the most severe form of nephritis and requires aggressive immunosuppressive treatment [2]. Despite improved treatment regimens, approximately 10% of all LN patients develop end-stage renal disease (ESRD) [1]. Several non-HLA susceptibility genes for SLE have been identified through candidate gene and genome wide association studies (GWAS). Among these, polymorphisms in signal transducer and activator of transcription 4 (*STAT4),* interferon regulatory factor 5 *(IRF5)*, the family with sequence similarity 167 member A-B lymphoid tyrosine kinase *(FAM167A-BLK)* locus, TNFAIP3 interacting protein 1 (*TNIP1*) and integrin-α_M_-integrin-α_X_ *(ITGAM-ITGAX)* display high signals of association that have been convincingly replicated [3]-[6].

The genetic background of LN has been less well elucidated and there are hitherto no GWAS published in LN [7]. Studies in Caucasian populations have demonstrated associations between LN and several gene polymorphisms including *STAT4*, *ITGAM*, *TNIP1, FAM167A-BLK,* programmed cell death 1 (*PDCD1)*, tumour necrosis factor alpha-induced protein 3 (*TNFAIP3)*, tumour necrosis factor superfamily 4 *(TNFSF4)*, apolipoprotein H (*APOH*, encoding beta-2-glycoprotein I), interleukin-6 *(IL-6)*, monocyte chemoattractant protein 1 *(MCP-1)*, Fcγ receptor *(FcγR)*, XK, Kell blood group complex subunit-related family member 6 *(XKR6)* and C-reactive protein *(CRP)* genes, though with inconsistent results [8]–[19]. Few studies have addressed the potential association between susceptibility genes, type of LN and renal outcome. Taylor and co-workers found an association between the single nucleotide polymorphism (SNP) rs7574865 in *STAT4* and severe nephritis, defined as histopathologic evidence of severe, progressive disease or ESRD [18]. An association between proliferative nephritis and SNPs in *FcγR3A* and *CRP* has also been demonstrated [10].

The aim of this study was to further elucidate the genetic component in LN using data on more than 5000 SNPs generated in our previous study on SLE [6]. In addition, the association between LN susceptibility genes and proliferative nephritis as well as the development of severe renal insufficiency was investigated.

## Materials and Methods

### Patients and controls

Cohort I consisted of 567 Swedish Caucasian patients with SLE and 512 controls. The patients originate from the rheumatology clinics in the Uppsala (n = 143) and Lund (n = 155) University Hospitals and the Karolinska University Hospital, Stockholm (n = 269), Sweden. The controls were individually matched for age, gender and area of residence and consisted of healthy blood donors from Uppsala (n = 132) and Lund (n = 91) while the controls from Stockholm (n = 289) were samples from the population-based Epidemiological Investigation of Rheumatoid Arthritis (EIRA) control cohort [20]. Cohort II consisted of 145 Swedish SLE patients of Caucasian origin from Linköping University Hospital, and 619 healthy Swedish blood donor controls. Five hundred and fifty two cases and 499 controls in cohort I and 144 cases and all controls in cohort II were previously described in a study by Wang et al [19]. All patients fulfilled the 1982 ACR criteria for SLE [21]. Clinical data was extracted from the patient files.

### Definition of lupus nephritis

Occurrence of LN was defined according to the ACR criteria and onset of nephritis was defined as the year of fulfilling ACR nephritis criteria. Renal biopsies were performed in 159 patients in cohort I, for which 152 histopathology results were available. In cohort II, 26 patients were subjected to renal biopsies, all with available histopathology results. LN according to the WHO classification system was verified in 160 patients (134 in cohort I and 26 in cohort II) [2], while in cohort I, 18 biopsies showed vascular changes including thrombotic microangiopathy (data not shown). Patients having WHO class III or IV at any time were defined as the proliferative nephritis group (Table? 1).

**Table 1:** Patient basic characteristics.

	Cohort I^a^				Cohort II^b^			
	All SLE patients	SLE with lupus nephritis	SLE without lupus nephritis	P^c^	All SLE patients	SLE with lupus nephritis	SLE without lupus nephritis	P^c^
Number of patients (%)	567	195 (34.4)	372 (65.6)		145	35 (24.1)	110 (75.9)	
Number of females (%)	501 (88.4)	159 (81.5)	342 (91.9)	0.0002	129 (89.0)	27 (77.1)	102 (92.7)	0.01
Age at SLE diagnosis, years (mean, sd)	34.4±14.9	30.1±14.6	36.5±14.5	6.4×10^−8^	39.1±17.8	29.6±13.7	42.2±18.0	3.7×10^−5^
Disease duration, years (mean, sd)	21.1±11.6	21.7±10.9	20.8±12.0	0.18	14.0±10.4	19.1±11.1	12.4±9.7	7.7×10^−4^
Age at nephritis onset (mean, sd)		34.0±15.4				33.0±14.1		
Number of ACR criteria (mean, sd)	5.7±1.4	6.3±1.5	5.4±1.2	6.4×10^−12^	4.9±1.1	5.9±1.3	4.6±0.8	2.3×10^−8^
Number of patients with proliferative nephritis^d^ (%)		92/152 (60.1)				20/26 (76.9)		
Number of patients with severe renal insufficiency^e^ (%)		28/190 (14.7)				3/35 (8.6)		

^a^ Uppsala, Stockholm and Lund, Sweden

^b^ Linköping, Sweden

^c^ Comparison between SLE with lupus nephritis and SLE without lupus nephritis. Frequencies compared with Chi square test and continuous variables with Mann-Whitney U-test.

^d^ WHO class III or IV on renal biopsy, according to the 1995 WHO classification system [2]. Biopsy data was not available from all patients.

^e^ Glomerular filtration rate <30 mL/min/1.73 m^2^ [22].

### Renal insufficiency outcome

The follow-up time from nephritis onset was until December 31^st^ 2009 (median 14 years, range 0–46). The glomerular filtration rate (GFR) was calculated at follow up with the Modification of Diet in Renal Disease (MDRD) formula. Renal function was classified according to the chronic kidney disease (CKD) system where stage 4 and 5 (GFR <30 mL/min/1.73 m^2^) were defined as severe renal insufficiency [22]. Patient characteristics are presented in Table? 1.

### Ethics statement

The study was conducted according to the Declaration of Helsinki. All patients from Stockholm, Lund and Linköping gave their verbal and written informed consent and the patients from Uppsala gave their verbal consent recorded in the patient files. The study and consent procedure was approved by the local ethics committees; the committee for research ethics at Karolinska hospital in Stockholm, the Regional Ethics Board Lund University, the Regional Ethics committee in Linköping and the Regional Ethical Review Board in Uppsala, Sweden. Clinical and laboratory data is coded and stored on paper and in a research database. According to all local ethical rules only the principal investigators have the identity key (i.e can trace the data back to the individual patient) for this database.

### Genotyping

All individuals in cohort I were genotyped on custom 12 k Illumina iSelect BeadArrays as previously described by Gateva *et al* [6] and had passed rigorous genotype quality control, including removal of population outliers. A total of 5676 SNPs remained after genotype quality control filters and exclusion of ancestry informative markers. The samples in cohort II were genotyped with a custom 384plex Illumina VeraCode GoldenGate assay (Illumina Inc, CA, USA). For this assay SNPs were excluded which did not conform to Hardy-Weinberg equilibrium (Chi-square test, p <0.01), had genotype call rates <90% or reproducibility <99.5% as determined by replicated genotyping of 5% of samples.

### Statistical analysis

Patient characteristics were compared between SLE patients with and without LN. Frequencies were compared with Chi square test and continuous variables with Mann-Whitney U-test and one-way ANOVA using Statistica® software version 10. Allele frequencies in case-control and case-only analyses were compared using Fisher's exact test, conditioning on the matched pairs and meta-analysis was performed with the Cochran-Mantel-Haenszel test and linkage disequilibrium (LD) between SNPs was obtained from the genotypes of 512 controls, using the PLINK software version 1.07 [23]. Association analyses including age or disease duration and gender as covariates were performed using logistic regression in PLINK. A quantile-quantile (Q-Q) plot of observed versus expected p-values was generated from the results of the LN case-control study in cohort I and statistical power was estimated using the Software Quanto v1.2.4 (http://hydra.usc.edu/gxe) assuming a log-additive model. For the case-only analysis of 13 SNPs in nine genes where the two SNPs in each of *STAT4*, *IRF5*, *TNIP1* and *BLK* are in high LD, a Bonferroni correction for nine loci was performed and p <0.006 was considered significant. In the case-control and case-only meta-analysis of four SNPs in cohort I and II, the significant Bonferroni corrected p-value was <0.0125. Unadjusted p-values are presented.

## Results

### Patients

The LN patients in both cohorts were significantly more often men, younger at disease onset and fulfilled more ACR criteria compared with the SLE patients without nephritis (Table? 1). There was no difference in disease duration between patients with or without LN in cohort I whereas in cohort II, patients with LN had significantly longer disease duration compared with SLE patients without nephritis. The renal biopsies showed a proliferative nephritis in 92/152 (60.1%) in cohort I and 20/26 (76.9%) in cohort II [2]. A total of 31 patients, 28 (14.7%) in cohort I and 3 (8.6%) in cohort II had developed severe renal insufficiency at follow up (Table? 1). In both cohorts, patients with severe renal insufficiency had a significantly longer disease duration compared with SLE patients without renal insufficiency: in cohort I 28.0±11.0 versus 20.7±11.5 years (p = 7.6×10^−4^), and in cohort II 27.3±7.7 versus 13.7±10.3 years, (p = 0.03) (data not shown). Proliferative nephritis was the most frequent cause of developing severe renal insufficiency. Out of the 31 patients with severe renal insufficiency at follow up, 24 had undergone a previous renal biopsy where 12 (50%) of the patients had a proliferative nephritis. Conversely, of the 112 patients with proliferative nephritis, outcome data was available for 110. The 12 patients whose outcome was severe renal insufficiency constituted 11% of all patients with proliferative nephritis (data not shown).

### Case-control association with lupus nephritis

In cohort I, we performed a LN case versus healthy control association analysis. The strongest signals of association were detected for four highly linked SNPs in *STAT4*; rs11889341, rs7574865, rs7568275 and rs7582694 (r^2^ = 0.98), with p-values reaching genome wide significance (p <5×10^−8^) (Figure? 1). The most significant SNP in *STAT4* was rs11889341 with p = 3.7×10^−9^, odds ratio (OR) 2.20 (95% confidence interval (CI) 1.70–2.84). Strong signals of association with LN were also detected for two nearly perfectly linked SNPs in *IRF5*, (rs2070197 and rs10488631, r^2^≈1.0) and the *HLA-DR3* marker SNP rs3135394, all p <1×10^−4^. In addition, SNPs in the postmeiotic segregation increased 2 *(PMS2)*, *TNIP1*, caspase recruitment domain family, member 11 (*CARD11), ITGAM, BLK* and interleukin-1 receptor-associated kinase 1 (*IRAK1)* genes were associated with LN with p-values <0.001 and OR between 1.53 and 2.21 (Table? 2, Table S1 and Figure? 1). Table? 2 lists the best SNP in each of the nine genes that displayed an association with LN with p <0.001 and for *STAT4*, *IRF5*, *TNIP1* and *BLK* also the SNPs used for further meta-analysis, see below. A Q-Q plot of the results from the LN versus healthy controls association analysis shows p-values deviating from the expected distribution indicating the presence of true associations (Figure S1).

**Figure 1:**
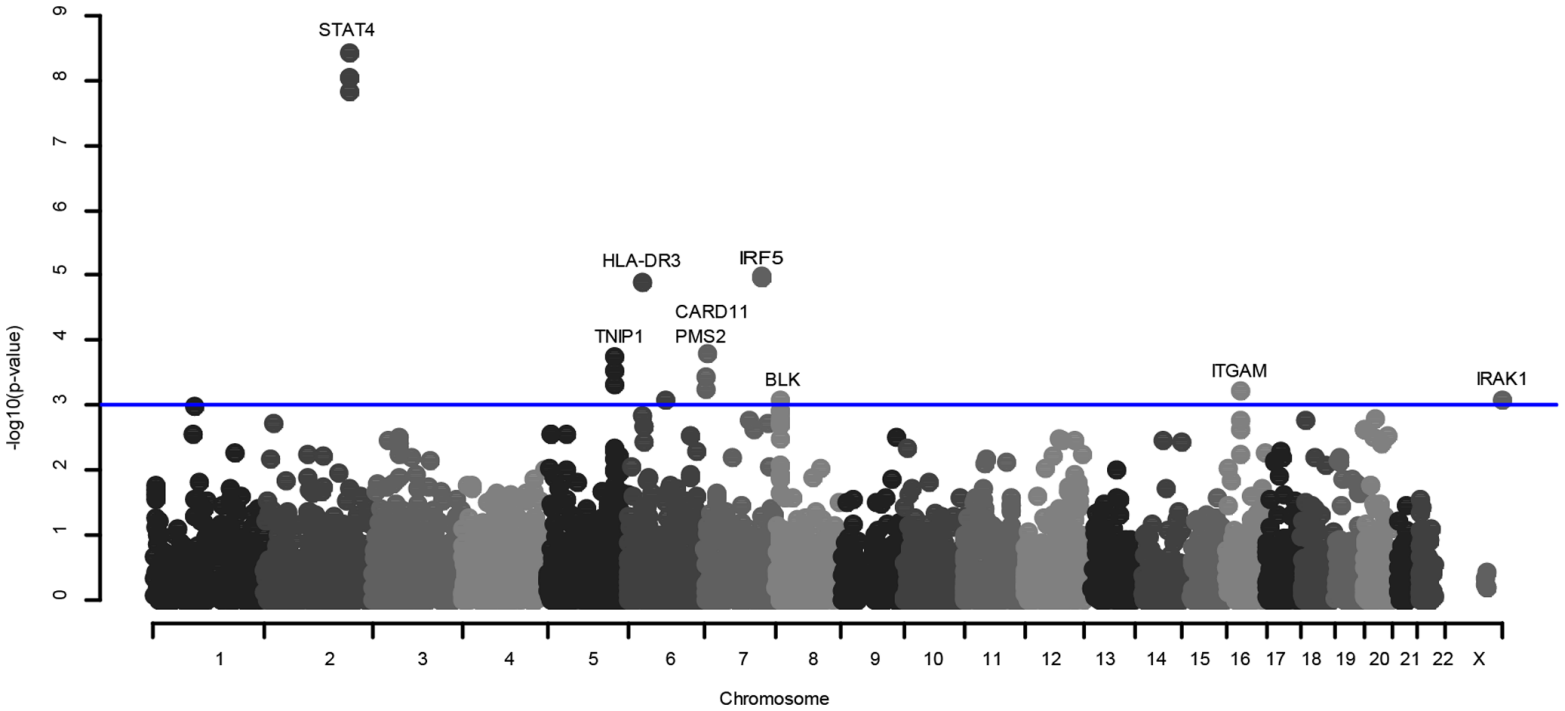
Association of 5676 SNPs to lupus nephritis in case-control analysis of 195 patients. Results from the association analysis of 5676 SNPs in 195 patients with lupus nephritis and 512 healthy controls in cohort I. The negative logarithm of the p-value is plotted against the chromosomal location. The line represent associations with p<0.001 and the nine genes associated with p<0.001 are indicated. The *STAT4* SNPs rs11889341, rs7574865, rs7568275 and rs7582694 have an r^2^ = 0.98 calculated from the 512 controls.

**Table 2:** Case-control association analysis in cohort I^a^. The best SNPs in genes associated with lupus nephritis with p <0.001 are shown.

			Lupus nephritis				Proliferative nephritis^b^				Severe renal insufficiency^c^				All SLE Cases			
			n = 195				n = 92				n = 28				n = 567			
Gene	Chr	SNP	P^e^	OR (95% CI)^e^	Padj^f^	OR (95% CI)adj^f^	P^e^	OR (95% CI)^e^	Padj^f^	OR (95% CI)adj^f^	P^e^	OR (95% CI)^e^	Padj^f^	OR (95% CI)adj^f^	P^e^	OR (95% CI)^e^	Padj^f^	OR (95% CI)adj^f^
STAT4	2	rs11889341	3.7×10^−9^	2.20 (1.70–2.84)	1.3×10^−6^	2.10 (1.56–2.84)	3.7×10^−7^	2.44 (1.75–3.40)	1.5×10^−5^	2.44 (1.63–3.65)	7.6×10^−6^	3.61 (2.09–6.23)	2.4×10^−5^	4.05 (2.12–7.73)	1.5×10^−11^	1.95 (1.60–2.37)	1.1×10^−8^	1.85 (1.50–2.28)
		rs7582694	1.5×10^−8^	2.12 (1.64–2.74)	3.2×10^−6^	2.04 (1.51–2.76)	2.9×10^−6^	2.27 (1.63–3.17)	5.0×10^−5^	2.30 (1.54–3.44)	1.0×10^−5^	3.52 (2.04–6.08)	2.9×10^−5^	3.93 (2.07–7.47)	4.6×10^−11^	1.92 (1.58–2.33)	1.6×10^−8^	1.83 (1.48–2.26)
IRF5	7	rs2070197	1.0×10^−5^	2.00 (1.48–2.71)	1.4×10^−5^	2.14 (1.52–3.01)	1.3×10^−4^	2.21 (1.50–3.25)	4.5×10^−5^	2.68 (1.67–4.30)	7.1×10^−3^	2.53 (1.36–4.71)	1.5×10^−3^	3.02 (1.53–6.00)	1.0×10^−9^	2.03 (1.61–2.56)	3.1×10^−8^	1.98 (1.55–2.51)
		rs10488631	1.1×10^−5^	1.99 (1.47–2.69)	1.6×10^−5^	2.13 (1.51–3.00)	1.4×10^−4^	2.18 (1.48–3.21)	5.5×10^−5^	2.65 (1.65–4.25)	7.1×10^−3^	2.54 (1.37–4.72)	1.5×10^−3^	3.03 (1.53–6.00)	1.0×10^−9^	2.03 (1.61–2.56)	3.1×10^−8^	1.98 (1.55–2.52)
HLA-DR3^d^	6	rs3135394	1.3×10^−5^	1.95 (1.45–2.62)	4.2×10^−5^	2.07 (1.46–2.93)	9.7×10^−5^	2.17 (1.49–3.16)	1.3×10^−4^	2.45 (1.55–3.88)	0.11	1.72 (0.89–3.34)	0.081	1.88 (0.92–3.81)	2.8×10^−10^	2.03 (1.62–2.54)	3.5×10^−10^	2.22 (1.73–2.84)
PMS2	7	rs1860460	1.6×10^−4^	1.67 (1.26–2.17)	3.6×10^−4^	1.79 (1.30–2.46)	0.031	1.49 (1.04–2.17)	0.061	1.49 (0.98–2.26)	0.77	1.12 (0.62–2.00)	0.55	1.22 (0.63–1.61)	4.7×10^−3^	1.30 (1.09–1.56)	4.1×10^−3^	1.35 (1.10–1.65)
TNIP1	5	rs6889239	1.8×10^−4^	1.62 (1.26–2.07)	2.6×10^−4^	1.70 (1.28–2.26)	9.2×10^−3^	1.58 (1.13–2.20)	0.01	1.66 (1.13–2.45)	0.53	1.23 (0.68–2.21)	0.27	1.42 (0.77–2.64)	3.8×10^−3^	1.32 (1.10–1.60)	4.0×10^−3^	1.34 (1.10–1.64)
		rs7708392	3.0×10^−4^	1.60 (1.25–2.05)	3.7×10^−4^	1.68 (1.26–2.23)	0.012	1.54 (1.11–2.15)	0.016	1.61 (1.09–2.38)	0.53	1.23 (0.68–2.21)	0.27	1.42 (0.77–2.64)	4.4×10^−3^	1.31 (1.09–1.58)	5.3×10^−3^	1.33 (1.09–1.63)
CARD11	7	rs17834873	3.7×10^−4^	2.21 (1.41–3.45)	1.4×10^−3^	2.24 (1.37–3.67)	1.5×10^−3^	2.86 (1.41–5.56)	6.4×10^−3^	2.84 (1.34–6.01)	0.14	2.47 (0.76–8.04)	0.17	2.32 (0.71–7.62)	7.0×10^−5^	1.79 (1.35–2.38)	4.5×10^−4^	1.74 (1.28–2.37)
ITGAM	16	rs1143679	5.9×10^−4^	1.82 (1.30–2.56)	6.7×10^−3^	1.70 (1.16–2.49)	0.027	1.71 (1.09–2.67)	0.010	1.97 (1.17–3.31)	0.17	1.75 (0.83–3.68)	0.17	1.70 (0.80–3.62)	1.6×10^−6^	1.86 (1.44–2.41)	2.1×10^−4^	1.67 (1.27–2.20)
BLK	8	rs922483	8.3×10^−4^	1.53 (1.19–1.95)	1.9×10^−3^	1.55 (1.18–2.05)	2.2×10^−3^	1.68 (1.21–2.32)	2.8×10^−3^	1.76 (1.22–2.55)	0.015	2.01 (1.17–3.47)	7.8×10^−3^	2.26 (1.24–4.11)	3.5×10^−3^	1.31 (1.10–1.58)	6.3×10^−3^	1.31 (1.08–1.59)
		rs13277113	1.7×10^−3^	1.51 (1.18–1.95)	5.0×10^−3^	1.51 (1.13–2.01)	8.2×10^−3^	1.59 (1.14–2.23)	0.018	1.59 (1.08–2.34)	0.012	2.05 (1.18–3.55)	0.013	2.11 (1.17–3.81)	7.8×10^−3^	1.30 (1.08–1.57)	0.012	1.30 (1.06–1.59)
IRAK1	23	rs1059702	8.7×10^−4^	1.78 (1.28–2.46)	NA	NA	2.7×10^−3^	1.97 (1.29–3.00)	NA	NA	0.36	1.48 (0.67–3.24)	NA	NA	5.3×10^−3^	1.43 (1.11–1.84)	NA	NA

The best SNP in each gene is shown and for STAT4, IRF5, TNIP1 and BLK also the SNPs used for meta-analysis, marked in bold; STAT4 rs11889341, rs7582694 r^2^ = 0.98, IRF5 rs2070197, rs10488631 r^2^≈1.00, TNIP1 rs7708392, rs6889239 r^2^≈1.00 and BLK rs922483, rs13277113 r^2^ = 0.87 calculated in 512 Swedish controls. OR: odds ratio, CI: confidence interval, NA: not available.

^a^ Uppsala, Stockholm and Lund, Sweden, n = 567 SLE cases, n = 512 controls

^b^ WHO class III or IV on renal biopsy, according to the 1995 WHO classification system [2].

^c^ Glomerular filtration rate <30 mL/min/1.73 m^2^ [22].

^d^ rs3135394 has an r^2^ = 0.87 with the HLA*DR3 (DRB1*0301) allele [6].

^e^ Unadjusted p-value and OR for differences in allele frequencies between patients and controls.

^f^ Adjusted p-value and OR from logistic regression analysis including age and gender as covariates. Number of cases and controls; lupus nephritis, n = 194, proliferative nephritis, n = 91, severe renal insufficiency, n = 28, SLE, n = 566, controls, n = 504.

### Case-control association with proliferative nephritis and outcome

The nine genes that had shown an association with LN in cohort I with p <0.001 were further studied. First association analysis of patients with proliferative nephritis (n = 92) versus healthy controls was performed. The *STAT4*, *IRF5* and *HLA-DR3* SNP proxy were all found to be associated with proliferative nephritis, with p <0.001, OR between 2.17 and 2.44 (Table? 2). Next the association between these genes and the outcome measure severe renal insufficiency was analyzed. Despite the low number of cases, LN patients who had developed severe renal insufficiency (n = 28) displayed signals of association with *STAT4* with p = 7.6×10^−6^ and OR 3.61 (95% CI 2.09–6.23) (Table? 2). When adjusting for age and gender, the effect measures (OR, 95% CI) were largely unchanged (Table? 2) and conditioning on the matched pairs yielded analogous results (data not shown).

### Case-only sub-phenotype analysis

Next a case-only association analysis was performed of the 13 SNPs in the nine genes that were associated with LN in cohort I. The risk allele frequencies were compared between patients with or without LN, proliferative nephritis and severe renal insufficiency, respectively. A nominal association was found with LN for SNPs in the *PMS2* and *TNIP1* genes (p <0.05) while there were no associations with proliferative nephritis. Signals of association between *STAT4* and the development of severe renal insufficiency were detected, with a p-value of 0.02, OR 1.91 (95% CI 1.11–3.28) for SNP rs11889341 (Table S2). However, correcting for the nine loci analysed, none of the associations remained significant. There was 64% power to detect an association between the *STAT4* risk allele rs11889341 and severe renal insufficiency (Table S3).

### Meta-analysis

To evaluate the robustness of our findings we used data from 145 patients with SLE from an additional Swedish cohort (cohort II) where detailed renal data were available (Table? 1) and 619 healthy Swedish controls. The *STAT4, IRF5*, *TNIP1* and *BLK* genes had been genotyped in cohort II. The SNPs genotyped in both cohort I and II, used for meta-analysis, were *STAT4* rs7582694 (r^2^ = 0.98 with rs11889341), *IRF5* rs10488631 (r^2^≈1.00 with rs2070197), *TNIP1* rs7708392 (r^2^≈1.00 with rs6889239) and *BLK* rs13277113 (r^2^ = 0.87 with rs922483) (Table? 2). In the LN case versus healthy controls (n = 1131) meta-analysis, the SNPs in *STAT4* and *IRF5* were associated with LN with genome wide significance (p <5×10^−8^). An association between the *STAT4* SNP rs7582694 and severe renal insufficiency at the genome wide significance level was also detected (Table? 3). In the case-only meta-analysis of the two cohorts there was a significant association between the *STAT4* SNP rs7582694 and severe renal insufficiency with p = 1.6×10^−3^, OR 2.22 (95% CI 1.34–3.70) (Table? 4).

**Table 3:** Case-control meta-analysis of cohort I^a^ and cohort II^b^ in a total of 712 SLE cases and 1131 controls.

		Lupus nephritis				Proliferative nephritis^c^				Severe renal insufficiency^d^				SLE			
		n = 230				n = 112				n = 31				n = 712			
Gene	SNP	P^e^	OR (95% CI)^e^	Padj^f^	OR (95% CI)adj^f^	P^e^	OR (95% CI)^e^	Padj^f^	OR (95% CI)adj^f^	P^e^	OR (95% CI)^e^	Padj^f^	OR (95% CI)adj^f^	P^e^	OR (95% CI)^e^	Padj^f^	OR (95% CI)adj^f^
STAT4	rs7582694	*2.4×10^−9^*	1.99 (1.59–2.50)	*5.7×10^−6^*	1.85 (1.42–2.41)	*6.0×10^−7^*	2.11 (1.57–2.84)	*1.1×10^−4^*	1.99 (1.40–2.83)	*2.3×10^−8^*	4.00 (2.38–6.72)	*2.9×10^−5^*	3.93 (2.07–7.47)	*1.0×10^−9^*	1.65 (1.40–1.93)	*6.2×10^−7^*	1.56 (1.31–1.86)
IRF5	rs10488631	*2.9×10^−8^*	2.10 (1.61–2.74)	*1.4×10^−7^*	2.28 (1.68–3.09)	*2.3×10^−6^*	2.24 (1.59–3.16)	*5.6×10^−6^*	2.61 (1.72–3.94)	*3.2×10^−5^*	3.11 (1.77–5.46)	*1.5×10^−3^*	3.03 (1.53–6.00)	*1.6×10^−15^*	2.13 (1.76–2.57)	*6.3×10^−14^*	2.20 (1.79–2.70)
TNIP1	rs7708392	*1.2×10^−4^*	1.55 (1.24–1.94)	*7.3×10^−4^*	1.56 (1.20–2.01)	*5.2×10^−3^*	1.53 (1.13–2.06)	0.022	1.50 (1.06–2.12)	0.43	1.25 (0.72–2.17)	0.27	1.42 (0.77–2.64)	*3.4×10^−4^*	1.33 (1.14–1.55)	*3.8×10^−3^*	1.28 (1.08–1.52)
BLK	rs13277113	*3.0×10^−3^*	1.42 (1.13–1.78)	*6.8×10^−3^*	1.43 (1.10–1.85)	*3.4×10^−3^*	1.56 (1.16–2.11)	*5.9×10^−3^*	1.62 (1.15–2.28)	*0.009*	1.99 (1.18–3.35)	0.013	2.11 (1.17–3.81)	0.017	1.22 (1.04–1.43)	0.025	1.22 (1.02–1.44)

^a^ Uppsala, Stockholm, Lund, n = 567 cases, n = 512 controls.

^b^ Linköping, n = 145 cases, n = 619 controls.

^c^ WHO class III or IV on renal biopsy, according to the 1995 WHO classification system [2]. Biopsies available in 178 patients.

^d^ Glomerular filtration rate <30 mL/min/1.73 m^2^ [22]. Data available for 225 patients.

^e^ Unadjusted combined p-value and OR calculated using Cochran-Mantel Haenszel chi-square test. P-values remaining significant after Bonferroni correction for 4 tested SNPs are in italic.

^f^ Adjusted p-value and OR from meta-analysis of logistic regression results including age and gender as covariates. Number of cases and controls; lupus nephritis, n = 229, proliferative nephritis, n = 111, severe renal insufficiency, n = 31, SLE, n = 711, controls, n = 960.

**Table 4:** Case-only meta-analysis of cohort I^a^ and cohort II^b^ in a total of 712 SLE cases.

			Lupus nephritis				Proliferative nephritis^c^				Severe renal insufficiency^d^			
			n = 230				n = 112				n = 31			
Gene	Chr	SNP	P^e^	OR (95% CI)^e^	Padj^h^	OR (95% CI) adj^h^	P^f^	OR (95% CI)^f^	Padj^h^	OR (95% CI) adj^h^	P^g^	OR (95% CI)^g^	Padj^h^	OR (95% CI) adj^h^
STAT4	2	rs7582694	0.11	1.21 (0.96–1.54)	0.22	1.16 (0.91–1.49)	0.11	1.28 (0.95–1.72)	0.20	1.23 (0.90–1.679	*1.6×10^−3^*	2.22 (1.34–3.70)	0.020	1.91 (1.11–3.30)
IRF5	7	rs10488631	0.98	1.00 (0.76–1.30)	0.94	1.01 (0.76–1.34)	0.61	1.09 (0.78–1.53)	0.72	1.07 (0.75–1.53)	0.13	1.53 (0.88–2.66)	0.13	1.62 (0.87–3.01)
TNIP1	5	rs7708392	0.037	1.29 (1.02–1.63)	0.11	1.23 (0.95–1.59)	0.23	1.21 (0.89–1.63)	0.30	1.19 (0.86–1.64)	0.83	0.94 (0.54–1.63)	0.61	0.86 (0.48–1.54)
BLK	8	rs13277113	0.10	1.23 (0.96–1.56)	0.11	1.23 (0.95–1.58)	0.062	1.34 (0.99–1.82)	0.072	1.34 (0.97–1.83)	0.069	1.62 (0.96–2.73)	0.12	1.52 (0.89–2.60)

^a^ Uppsala, Stockholm, Lund, n = 567 cases.

^b^ Linköping, n = 145 cases.

^c^ WHO class III or IV on renal biopsy, according to the 1995 WHO classification system [2].

^d^ Glomerular filtration rate <30 mL/min/1.73 m^2^ [22].

^e^ Unadjusted p-value and OR for difference in allele frequencies between patients with (n = 230) and without (n = 482) lupus nephritis.

^f^ Unadjusted p-value and OR for difference in allele frequencies between patients with proliferative nephritis (n = 112) and SLE without proliferative nephritis (n =  548; SLE without LN, n = 482 and LN other than proliferative, n = 66).

^g^ Unadjusted p-value and OR for difference in allele frequencies between patients with severe renal insufficiency (n = 31) and SLE without severe renal insufficiency (n = 676; SLE without LN, n = 482 and LN without severe renal insufficiency at follow-up, n = 194). P-value remaining significant after Bonferroni correction for 4 tested SNPs is in italic.

^h^ Adjusted p-value and OR from meta-analysis of logistic regression results including disease duration and gender as covariates. Number of cases in each analysis; LN/non-LN: 225/481, proliferative nephritis/non-proliferative nephritis: 109/545, severe renal insufficiency/non-severe renal insufficiency: 31/670.

LN patients with severe renal insufficiency had a longer disease duration compared with SLE patients without this adverse outcome. We therefore investigated whether there were any differences in disease duration between the three genotypes in *STAT4, IRF5, TNIP1* and *BLK*. There were no differences in disease duration between patients homozygous for the risk allele (minor allele), heterozygous or homozygous for the non-risk allele (major allele) for *STAT4, IRF5, TNIP1* or *BLK* when comparing all SLE patients or LN patients alone (Table S4). In both cohorts there were a significantly higher proportion of men in the SLE with LN group, compared with the SLE without LN group (Table? 1). However, there were no differences in genotypes between the genders for the risk alleles in *STAT4, IRF5, TNIP1* or *BLK* (Table S5). When adjusting for the potential confounders age and gender in the case-control analyses, the effect measures (OR, 95% CI) were largely unchanged. Finally, disease duration and gender were included as covariates in the case-only meta-analysis of the two cohorts. In this analysis the association between the *STAT4* SNP rs7582694 and severe renal insufficiency was no longer significant after correction for multiple analyses (p = 0.020, OR 1.91, 95% CI 1.11–3.30) (Table? 4).

## Discussion

Here we demonstrate that polymorphisms in the *STAT4* gene are associated with LN with genome wide significance in a LN case-versus-healthy controls analysis of Swedish SLE patients. Furthermore an association between the *STAT4* SNP rs7582694 and severe renal insufficiency was present in our case-only meta-analysis of two independent SLE cohorts. Although we were not able to detect a significant association with *STAT4* in our case-only analysis of LN versus non-LN SLE patients, possibly due to a lack of power, our results support previous observations that *STAT4* is particularly strongly associated with a more severe SLE phenotype with renal engagement [18]. In the study by Taylor and co-workers [18] an association between the *STAT4* SNP rs7574865, in strong LD (r^2^>0.99) with the SNP rs7582694 here studied, and LN was found in a case-control and case-only analysis. Here we extend the knowledge and present for the first time the association between *STAT4* and the severe form of proliferative nephritis in a case-control analysis. We also demonstrate for the first time in our case-only analysis, an association between *STAT4* and a worse outcome in terms of severe renal insufficiency defined as a GRF <30 mL/min/1.73 m^2^ at follow up.

The highly linked SNPs rs11889341, rs7574865, rs7568275 and rs7582694 (r^2^ = 0.98) in *STAT4*, which showed an association with LN in our case-control study, are all located in the large third intron. While there are no common coding *STAT4* SNPs in LD with these intronic SNPs, their possible functional role has been investigated. Allelic expression analysis have observed an overexpression of the *STAT4* risk allele (rs8179673, r^2^ = 0.95 to rs7582694) in human cells of mesenchymal origin but not in transformed B-cells, indicating that these intronic SNPs may regulate gene expression in different tissues [16]. There are several possible mechanisms by which STAT4 may contribute to the development of LN. The main STAT4 activating cytokines are interleukin 12 (IL-12) and IL-23 leading to Th1 and Th17 differentiation with IFN-γ and IL-17 production, which are key players in a pro-inflammatory immune response [24]. These pro-inflammatory properties are crucial in both the initiation and progression of the inflammatory renal disease process as reviewed in [25]. There is, in particular, increasing evidence implicating a role for IL-17 in LN pathogenesis. High levels of IL-17 at LN onset have been associated with a less favourable histopathological response to treatment and IL-17 producing cells have been detected in renal biopsies from LN patients [26], [27].

STAT4 also signals via the type I IFN receptor. SLE patients carrying the *STAT4* risk allele rs7574865 have an increased sensitivity to IFN-α signaling, measured as an increased expression of IFN-α regulated genes [28]. An increased activation of IFN stimulated genes (ISG) will promote the autoimmune process by activating a number of cells in the immune system as reviewed by Rönnblom *et al* in [29]. One such ISG is *TNFSF13B* that encodes B cell activating factor (BAFF)/B lymphocyte stimulator (BLyS) which promotes B cell differentiation and autoantibody production, including antibodies against double stranded DNA (anti-dsDNA) [30], [31]. Several investigators have also detected high signals of association between *STAT4* and anti-dsDNA autoantibodies [16], [18], [32], [33]. Consequently, risk-variants of *STAT4*, via several different effects in the immune system, could promote a progressive autoimmune process in the kidney, which ultimately may lead to renal failure.

Polymorphisms in *IRF5* display strong signals of association with SLE in populations of different ethnicities [5], [34]–[36]. No particular SLE phenotype has been associated with *IRF5* in case-only analyses but an association with the presence of anti-dsDNA antibodies has been demonstrated [32]. A case-control analysis in an Asian population demonstrated an association between LN and *IRF5*; however, this could represent the strong association with SLE per se [37]. Here we found the association with LN to be of similar strength as the association with SLE. This is in concordance with other studies where *IRF5* polymorphisms have been strongly associated with SLE regardless of disease phenotype [33], [38].

The *TNIP1* SNP rs7708392 has been associated with LN in case-control studies of Asian and Caucasian populations [19], [39]. In our previous case-only analysis of an SLE patient cohort largely overlapping with this study, a nominally significant association with LN was shown, notwithstanding multiple testing correction [19]. In this study we extended the investigation and explored the association with proliferative nephritis and renal outcome. However, no associations with these sub-phenotypes were shown. The *FAM167A-BLK* locus intergenic SNP rs13277113 was recently associated with LN in a case-only analysis [8] whereas previous studies have failed to find an association between this SNP and LN [3], [14]. In this study we did not detect any significant associations with BLK in our case-only analysis of LN, proliferative nephritis or renal outcome.

Cohort I was analysed for association with LN using a large set of SNPs. Interestingly, in the case-control analysis, the HLA-DR3 (DRB1*0301) SNP marker was not as strongly associated with LN as *STAT4*. While the *HLA-DR3* allele confers a 2-3 fold increased risk of SLE in Caucasian populations the role of the MHC in LN has been less well elucidated [7], [40]. One study found an association between an *HLA-DR3* SNP proxy and LN [33] whereas we detected similar minor allele frequencies for the HLA-DR3 (DRB1*0301) SNP proxy in LN, LN sub-phenotypes and all SLE cases (Table? 2).

An association between the SNP rs1143679 in *ITGAM* and LN has previously been demonstrated in both case-control and case-only analyses in patients of European and Asian ancestry with SLE [11], [14], [41]. Here we replicate the results in the LN case versus control analysis in cohort I. The association between LN or SLE and SNPs in the *PMS2* and *CARD11* genes, both located on chromosome 7, has hitherto not been reported. The *PMS2* gene encodes a DNA mismatch repair endonuclease and mutations have been associated with malignancies, in particular colorectal cancer [42]. Mutations in *CARD11* have been associated with diffuse large B cell lymphomas [43], the dominating lymphoma subtype in SLE [44]. The polymorphisms detected here in association with LN and SLE are intriguing and warrant further investigations. Finally, we found an association between *IRAK1* and LN in our case-control analysis while the case-only analysis displayed an unadjusted p-value of 0.05 for association with LN. Polymorphisms in *IRAK1* have previously been associated with SLE but the possible association with LN remains to be established [45].

The strength of this study is the availability of renal biopsies from the majority of patients as well as the longterm follow-up data on renal outcome. Approximately two thirds of our LN patients with available biopsies were diagnosed with a proliferative nephritis, which is linked to a less favourable renal outcome [46]. In this study 11% of the patients with proliferative nephritis had progressed to severe renal failure at follow up. This is in concordance with previous reports where approximately 10% of all patients with LN develop ESRD [1]. Only half of our patients with severe renal insufficiency where a previous biopsy was available had progressed from a proliferative nephritis. We therefore conclude that the two groups proliferative nephritis and severe renal insufficiency are not completely overlapping and that the case-only association between *STAT4* and severe renal insufficiency cannot be explained by an association with proliferative nephritis.

Renal biopsies had not been performed in 36/195 (18%) of the LN patients and 7 histopathology results could not be retrieved in cohort I, while in cohort II 9/35 (26%) of the LN patients had not undergone a biopsy. There are various reasons for not performing a renal biopsy, including a bleeding diathesis, uncontrolled hypertension, patients' or physicians' choice or for practical reasons. It is also possible that patients presenting with a clinically milder nephritis are less likely to undergo a biopsy. LN patients without biopsies were excluded from the case-only analysis of proliferative nephritis versus non-proliferative nephritis and the results presented are based on available data. However, the lack of complete biopsy data from all our LN patients has decreased the power to detect genetic associations with the sub-group proliferative nephritis.

A further weakness of our study is the low power to detect significant differences in the LN or renal outcome case-only analyses. The power to detect a possible association between *STAT4* and LN in our case-only analysis of cohort I was only 22% and for *TNIP1* 62% (Table S2). We had 62% power to detect an association between the *STAT4* risk allele rs7582694 and severe renal insufficiency in our case-only analysis of cohort I. However, the addition of cohort II increased the strength of the association between *STAT4* and severe renal insufficiency. Unfortunately, detailed clinical data on renal biopsies and outcome is not readily available from all centres, which has limited the inclusion of additional cohorts in this study.

One can hypothesize that there is a genetic contribution to the type of nephritis an individual patient will develop as well as to outcome, as here demonstrated. Males with SLE are more likely to develop LN. We found no differences in genotypes between the genders and correcting the case-control analyses for age and gender did not affect the OR for association. We therefore conclude that in the case-control analyses, the genetic associations here presented are with LN, proliferative nephritis and severe renal insufficiency *per se*, regardless of age and gender. LN patients with severe renal insufficiency have a longer disease duration compared with patients without such adverse outcome. We found no differences in disease duration between the genotypes for *STAT4, IRF5, TNIP1* and *BLK*. While adjusting for disease duration and gender, the association between *STAT4* and severe renal insufficiency did not remain significant after Bonferroni correction. However, we conclude that in this study, the *STAT4* risk allele is associated with an adverse renal outcome and not with disease duration or gender. There are many reasons for an unfavourable outcome, including renal atherosclerosis, but certain susceptibility genes may contribute to a worse prognosis. Interestingly, the *STAT4* risk allele has been shown to be a risk factor for stroke and anti-phospholipid antibodies in patients with SLE [47].

In conclusion, this LN case versus healthy control study has demonstrated an association between *STAT4* and LN with genome wide significance. In the case-only meta-analysis the *STAT4* risk allele displayed signals of association with a poor renal outcome with severe renal insufficiency. Future studies will try to define the precise role for this *STAT4* genetic variant in LN pathogenesis.

## Supporting Information

Figure S1 (DOCX)

Table S1 **Risk allele frequencies in cohort I**
(DOCX)

Table S2 **Case-only association analysis in cohort I**
(DOCX)

Table S3 **Power calculations in case-only analysis of cohort I**
(DOCX)

Table S4 **Disease duration by genotype**
(DOCX)

Table S5 **Genotype by gender**
(DOCX)
