# Supplementary material for: Association of STAT4 Polymorphism with Severe Renal Insufficiency in Lupus Nephritis
Source: PLoS One. 2013 Dec 27;8(12):e84450. doi: 10.1371/journal.pone.0084450 (PMC3873995; doi:10.1371/journal.pone.0084450)
Supplement: Figure S1 — (DOCX) [file pone.0084450.s001.docx]

**Figure S1. Q-Q plot of results from lupus nephritis versus controls association analysis in cohort I**

Q-Q plot of association results from lupus nephritis versus healthy controls analysis of 5676 SNPs in cohort I (n=195 lupus nephritis cases, n=512 controls). The observed P-value for each SNP is plotted against the theoretical distribution. The deviation from the null distribution suggests the presence of true associations in the observed data.
