# Supplementary material for: Association of STAT4 Polymorphism with Severe Renal Insufficiency in Lupus Nephritis
Source: PLoS One. 2013 Dec 27;8(12):e84450. doi: 10.1371/journal.pone.0084450 (PMC3873995; doi:10.1371/journal.pone.0084450)
Supplement: Table S1 — Risk allele frequencies in cohort I (DOCX) [file pone.0084450.s002.docx]

**Table S1. Risk allele frequencies in the case-control association analysis in cohort I^a^. The best SNPs in genes associated with lupus nephritis with p < 0.001 are shown**

|  |  |  |  | **Controls** | **Lupus nephritis** | **Proliferative nephritis^b^** | **Severe renal insufficiency^c^** | **All SLE Cases** |
| --- | --- | --- | --- | --- | --- | --- | --- | --- |
|  |  |  |  | **n=512** | **n=195** | **n=92** | **n=28** | **n=567** |
| **Gene** | **Chr** | **SNP** | **Risk allele** | **RAF** | **RAF** | **RAF** | **RAF** | **RAF** |
| STAT4 | 2 | rs11889341 | T | 0.21 | 0.36 | 0.39 | 0.48 | 0.33 |
|  |  | **rs7582694** | C | 0.21 | 0.36 | 0.38 | 0.48 | 0.34 |
| IRF5 | 7 | rs2070197 | C | 0.13 | 0.22 | 0.24 | 0.27 | 0.23 |
|  |  | **rs10488631** | C | 0.13 | 0.22 | 0.24 | 0.27 | 0.23 |
| HLA-DR3^d^ | 6 | rs3135394 | C | 0.14 | 0.24 | 0.26 | 0.21 | 0.24 |
| PMS2 | 7 | rs1860460 | T | 0.67 | 0.77 | 0.76 | 0.70 | 0.73 |
| TNIP1 | 5 | rs6889239 | C | 0.26 | 0.36 | 0.36 | 0.31 | 0.32 |
|  |  | **rs7708392** | C | 0.26 | 0.36 | 0.35 | 0.30 | 0.32 |
| CARD11 | 7 | rs17834873 | A | 0.87 | 0.94 | 0.95 | 0.94 | 0.92 |
| ITGAM | 16 | rs1143679 | A | 0.10 | 0.17 | 0.16 | 0.16 | 0.17 |
| BLK | 8 | rs922483 | T | 0.29 | 0.38 | 0.40 | 0.45 | 0.34 |
|  |  | **rs13277113** | A | 0.25 | 0.34 | 0.35 | 0.41 | 0.31 |
| IRAK1 | 23 | rs1059702 | T | 0.12 | 0.20 | 0.21 | 0.17 | 0.17 |

The best SNP in each gene is shown and for STAT4, IRF5, TNIP1 and BLK also the SNPs used for meta-analysis, marked in bold; STAT4 rs11889341, rs7582694 r^2^=0.98, IRF5 rs2070197, rs10488631 r^2^≈1.00, TNIP1 rs7708392, rs6889239 r^2^≈1.00 and BLK rs922483, rs13277113 r^2^=0.87 calculated in 512 Swedish controls. RAF: risk allele frequency.

^a^Uppsala, Stockholm and Lund, Sweden
^b^WHO class III or IV on renal biopsy, according to the 1995 WHO classification system [[1](#_ENREF_1)].
^c^Glomerular filtration rate <30 mL/min/1.73m^2^ [[2](#_ENREF_2)].
^d^rs3135394 has an r^2^ = 0.87 with the HLA*DR3 (DRB1*0301) allele [[3](#_ENREF_3)].
