# Supplementary material for: Association of STAT4 Polymorphism with Severe Renal Insufficiency in Lupus Nephritis
Source: PLoS One. 2013 Dec 27;8(12):e84450. doi: 10.1371/journal.pone.0084450 (PMC3873995; doi:10.1371/journal.pone.0084450)
Supplement: Table S2 — Case-only association analysis in cohort I (DOCX) [file pone.0084450.s003.docx]

**Table S2. Case-only association analysis in cohort I^a^**

|  |  |  | **Lupus Nephritis** | | | | **Proliferative nephritis^b^** | | | | **Severe renal insufficiency^c^** | | | |
| --- | --- | --- | --- | --- | --- | --- | --- | --- | --- | --- | --- | --- | --- | --- |
|  |  |  | **Pos** | **Neg^d^** |  |  | **Pos** | **Neg^e^** |  |  | **Pos** | **Neg^f^** |  |  |
|  |  |  | **n=195** | **n=372** |  |  | **n=92** | **n=432** |  |  | **n=28** | **n=534** |  |  |
| **Gene** | **Chr** | **SNP** | **RAF** | **RAF** | **P** | **OR (95% CI)** | **RAF** | **RAF** | **P** | **OR (95% CI)** | **RAF** | **RAF** | **P** | **OR (95% CI)** |
| STAT4 | 2 | rs11889341 | 0.36 | 0.32 | 0.16 | 1.20 (0.93-1.56) | 0.39 | 0.32 | 0.12 | 1.31 (0.94-1.82) | 0.48 | 0.33 | 0.020 | 1.91 (1.11-3.28) |
|  |  | **rs7582694** | 0.36 | 0.32 | 0.26 | 1.17 (0.90-1.51) | 0.38 | 0.33 | 0.23 | 1.23 (0.89-1.71) | 0.48 | 0.33 | 0.029 | 1.89 (1.10-3.25) |
| IRF5 | 7 | rs2070197 | 0.22 | 0.23 | 0.88 | 0.98 (0.73-1.31) | 0.24 | 0.22 | 0.63 | 1.11 (0.76-1.61) | 0.27 | 0.23 | 0.51 | 1.26 (0.69-2.31) |
|  |  | **rs10488631** | 0.22 | 0.23 | 0.88 | 0.97 (0.72-1.30) | 0.24 | 0.22 | 0.63 | 1.09 (0.75-1.59) | 0.27 | 0.22 | 0.51 | 1.26 (0.69-2.32) |
| HLA-DR3^j^ | 6 | rs3135394 | 0.24 | 0.25 | 0.72 | 0.94 (0.71-1.25) | 0.26 | 0.24 | 0.64 | 1.09 (0.75-1.57) | 0.21 | 0.25 | 0.75 | 0.84 (0.44-1.61) |
| PMS2 | 7 | rs1860460 | 0.77 | 0.70 | 0.014 | 1.44 (1.08-1.91) | 0.76 | 0.72 | 0.32 | 1.22 (0.85-1.75) | 0.70 | 0.70 | 0.54 | 0.85 (0.47-1.52) |
| TNIP1 | 5 | rs6889239 | 0.36 | 0.30 | 0.023 | 1.36 (1.05-1.77) | 0.36 | 0.31 | 0.19 | 1.26 (0.90-1.76) | 0.30 | 0.32 | 0.88 | 0.92 (0.51-1.65) |
|  |  | **rs7708392** | 0.36 | 0.29 | 0.022 | 1.36 (1.05-1.76) | 0.35 | 0.31 | 0.22 | 1.24 (0.88-1.73) | 0.30 | 0.32 | 0.88 | 0.93 (0.52-1.67) |
| CARD11 | 7 | rs17834873 | 0.94 | 0.92 | 0.24 | 1.35 (0.83-2.21) | 0.95 | 0.92 | 0.13 | 1.75 (0.85-3.57) | 0.94 | 0.92 | 0.79 | 1.41 (0.43-4.63) |
| ITGAM | 16 | rs1143679 | 0.17 | 0.17 | 0.93 | 0.97 (0.70-1.35) | 0.16 | 0.16 | 0.91 | 0.96 (0.62-1.48) | 0.16 | 0.17 | 1.00 | 0.94 (0.45-1.95) |
| BLK | 8 | rs922483 | 0.38 | 0.33 | 0.076 | 1.26 (0.98-1.63) | 0.40 | 0.33 | 0.060 | 1.37 (0.99-1.91) | 0.45 | 0.34 | 0.11 | 1.58 (0.92-2.72) |
|  |  | **rs13277113** | 0.34 | 0.29 | 0.077 | 1.27 (0.98-1.66) | 0.35 | 0.29 | 0.11 | 1.33 (0.95-1.86) | 0.41 | 0.30 | 0.10 | 1.64 (0.94-2.83) |
| IRAK1 | 23 | rs1059702 | 0.20 | 0.15 | 0.053 | 1.40 (1.00-1.95) | 0.21 | 0.16 | 0.065 | 1.49 (0.98-2.27) | 0.17 | 0.16 | 0.84 | 1.04 (0.48-2.26) |

The STAT4, IRF5, TNIP1 and BLK SNPs used for meta-analysis are marked in bold. RAF: risk allele frequency, OR: odds ratio, CI: confidence interval. Unadjusted p-value for differences in allele frequencies between patients with and without the phenotype/outcome.

^a^Uppsala, Stockholm and Lund, Sweden, n=567 patients with SLE.

^b^WHO class III or IV on renal biopsy, according to the 1995 WHO classification system [[1](#_ENREF_1)].

^c^Glomerular filtration rate <30 mL/min/1.73m^2^ [[2](#_ENREF_2)].
^d^SLE patients without LN (n=372).
^e^SLE without LN (n=372) and LN other than WHO class III-IV on renal biopsy (n=60). LN patients without available biopsy were excluded (n=43).

^f^SLE without LN (n=372) and LN without severe renal insufficiency in the follow-up (n=162). LN patients without available follow-up of renal function were excluded (n=5).

^g^rs3135394 has an r^2^ = 0.87 with the HLA*DR3 (DRB1*0301) allele [[3](#_ENREF_3)].
