# Supplementary material for: Association of STAT4 Polymorphism with Severe Renal Insufficiency in Lupus Nephritis
Source: PLoS One. 2013 Dec 27;8(12):e84450. doi: 10.1371/journal.pone.0084450 (PMC3873995; doi:10.1371/journal.pone.0084450)
Supplement: Table S4 — Disease duration by genotype (DOCX) [file pone.0084450.s005.docx]

**Table S4. Disease duration by genotype**

| **Gene** | **SNP** |  |  |  | **Genotypes** | | | **P**^b^ |
| --- | --- | --- | --- | --- | --- | --- | --- | --- |
| **STAT4** | **rs7582694** | **Genotypes** | | **CC/CG/GG** | **CC** | **CG** | **GG** |  |
| minor/major alleles | C/G | SLE patients, n= | | 706 | 78 | 303 | 325 |  |
|  |  |  | disease duration (years, sd) | 19.7 ± 11.8 | 22.2 ± 11.9 | 19.6 ± 11.9 | 19.2 ± 11.5 | 0.14 |
|  |  | Lupus nephritis patients, n= | | 229 | 27 | 110 | 92 |  |
|  |  |  | disease duration (years, sd) | 21.3 ± 10.9 | 23.3 ± 8.7 | 22.2 ± 11.0 | 19.6 ± 11.3 | 0.14 |
| **IRF5** | **rs10488631** | **Genotypes** | | **GG/GA/AA** | **GG** | **GA** | **AA** |  |
| minor/major alleles | G/A | SLE patients, n= | | 706 | 34 | 255 | 417 |  |
|  |  |  | disease duration (years, sd) | 19.7 ± 11.8 | 20.5 ± 9.3 | 19.6 ± 11.6 | 19.7 ± 12.0 | 0.92 |
|  |  | Lupus nephritis patients, n= | | 229 | 9 | 87 | 133 |  |
|  |  |  | disease duration (years, sd) | 21.3 ± 10.9 | 24.9 ± 7.0 | 21.0 ± 10.5 | 21.2 ± 11.4 | 0.60 |
| **TNIP1** | **rs7708392** | **Genotypes** | | **CC/CG/GG** | **CC** | **CG** | **GG** |  |
| minor/major alleles | C/G | SLE patients, n= | | 704 | 65 | 319 | 320 |  |
|  |  |  | disease duration (years, sd) | 19.7 ± 11.8 | 20.0 ± 13.1 | 20.0 ± 11.8 | 19.4 ± 11.5 | 0.81 |
|  |  | Lupus nephritis patients, n= | | 228 | 26 | 110 | 92 |  |
|  |  |  | disease duration (years, sd) | 21.3 ± 11.0 | 21.7 ± 11.0 | 21.3 ± 10.9 | 21.2 ± 11.0 | 0.98 |
| **BLK** | **rs13277113** | **Genotypes** | | **AA/AG/GG** | **AA** | **AG** | **GG** |  |
| minor/major alleles | A/G | SLE patients, n= | | 697 | 66 | 281 | 350 |  |
|  |  |  | disease duration (years, sd) | 19.7 ± 11.7 | 20.1 ± 11.8 | 20.3 ± 11.8 | 19.2 ± 11.6 | 0.54 |
|  |  | Lupus nephritis patients, n= | | 228 | 24 | 101 | 103 |  |
|  |  |  | disease duration (years, sd) | 21.3 ± 11.0 | 24.3 ± 8.8 | 21.6 ± 11.6 | 20.2 ± 10.7 | 0.24 |

^a^Disease duration for all analyses; onset of SLE until follow up. SLE; n=712, Lupus nephritis (LN); n=230. Data on disease duration was missing from 2 SLE patients; one with LN and one without LN. Genotype data was not available from all patients.

^b^P-value for the difference in disease duration between the three genotypes calculated with one-way ANOVA.
