# Supplementary material for: Association of STAT4 Polymorphism with Severe Renal Insufficiency in Lupus Nephritis
Source: PLoS One. 2013 Dec 27;8(12):e84450. doi: 10.1371/journal.pone.0084450 (PMC3873995; doi:10.1371/journal.pone.0084450)
Supplement: Table S5 — Genotype by gender (DOCX) [file pone.0084450.s006.docx]

**Table S5. Genotype by gender**

| **Gene** | **SNP** |  |  |  | **Genotypes** | | |
| --- | --- | --- | --- | --- | --- | --- | --- |
| **STAT4** | **rs7582694** | **Genotypes** | | **CC/CG/GG** | **CC** | **CG** | **GG** |
| minor/major | C/G | Females, n= | | 617 | 67 | 263 | 287 |
|  |  |  | *genotype frequency* |  | *0.11* | *0.43* | *0.47* |
|  |  | Males, n= | | 87 | 10 | 38 | 39 |
|  |  |  | *genotype frequency* |  | *0.11* | *0.44* | *0.45* |
|  |  | P-value^a^ | |  | 0.86 | 0.85 | 0.77 |
| **IRF5** | **rs10488631** | **Genotypes** | | **GG/GA/AA** | **GG** | **GA** | **AA** |
| minor/major | G/A | Females, n= | | 617 | 30 | 225 | 362 |
|  |  |  | *genotype frequency* |  | *0.05* | *0.36* | *0.59* |
|  |  | Males, n= | | 87 | 3 | 31 | 53 |
|  |  |  | *genotype frequency* |  | *0.03* | *0.36* | *0.61* |
|  |  | P-value^a^ | |  | 0.56 | 0.88 | 0.69 |
| **TNIP1** | **rs7708392** | **Genotypes** | | **CC/CG/GG** | **CC** | **CG** | **GG** |
| minor/major | C/G | Females, n= | | 615 | 54 | 278 | 283 |
|  |  |  | *genotype frequency* |  | *0.09* | *0.45* | *0.46* |
|  |  | Males, n= | | 87 | 10 | 39 | 38 |
|  |  |  | *genotype frequency* |  | *0.11* | *0.45* | *0.44* |
|  |  | P-value^a^ | |  | 0.41 | 0.95 | 0.68 |
| **BLK** | **rs13277113** | **Genotypes** | | **AA/AG/GG** | **AA** | **AG** | **GG** |
| minor/major | A/G | Females, n= | | 608 | 56 | 250 | 302 |
|  |  |  | *genotype frequency* |  | *0.09* | *0.41* | *0.50* |
|  |  | Males, n= | | 87 | 10 | 31 | 46 |
|  |  |  | *genotype frequency* |  | *0.11* | *0.36* | *0.53* |
|  |  | P-value^a^ | |  | 0.50 | 0.33 | 0.58 |

.

^a^P-value for the difference in genotype frequency between females and males for each genotype, calculated with Chi square test. Genotype data was not available from all patients.
